# Negative surgical exploration for CT-proven pneumoperitoneum: a diagnostic pitfall in suspected gastrointestinal perforation—a six-case series

**DOI:** 10.3389/fsurg.2026.1739471

**Published:** 2026-03-31

**Authors:** Min Chang Kang, Hayeon Lee, Haewon Lee, Hak-Jae Lee, Kyungwon Lee, Nam-Ryong Choi, Jay Jung, Eunhae Um, Hyunwoo Sun

**Affiliations:** 1 Department of Critical Care Medicine, Uijeongbu Eulji Medical Center, Eulji University College of Medicine, Uijeongbu, Republic of Korea; 2 Department of Surgery, Ilsan Paik Hospital, Inje University College of Medicine, Goyang, Republic of Korea

**Keywords:** acute care surgery, acute peritonitis, diagnostic decision-making, gastrointestinal perforation, negative surgical exploration, pneumoperitoneum

## Abstract

**Background:**

Computed tomography (CT) plays a vital role in diagnosing suspected gastrointestinal perforation (GIP), particularly in the presence of pneumoperitoneum and intra-abdominal fluid. Nevertheless, these radiologic findings do not invariably correspond to bowel perforation, and negative surgical exploration may occur. We conducted this study to characterize the clinical features and outcomes of patients who underwent emergency surgery for suspected GIP but had no perforation.

**Methods:**

We retrospectively reviewed consecutive non-trauma patients who underwent emergency surgical exploration during 2021–2025 for CT-confirmed pneumoperitoneum with fluid collection. Among 1,024 emergency surgical explorations (excluding appendectomies), 11 (1.1%) were classified as negative explorations, of which five cases without pneumoperitoneum were excluded, leaving six patients for analysis. We evaluated their clinical characteristics, laboratory findings, operative details, and 90-day outcomes.

**Results:**

The median age of the patients was 67.5 years [interquartile range (IQR): 58–77], and three patients (50%) were men. All patients presented with abdominal pain, with a median symptom duration of 84 h (IQR: 72–148). The median white blood cell count was 13.2 × 10^9^/L (IQR: 8.7–17.7), and the median C-reactive protein level was 23.6 mg/dL (IQR: 19.8–29.7). Final diagnoses included primary peritonitis (*n* = 1), sealed duodenal microperforation (*n* = 1), peritoneal abscess secondary to urinary tract infection (*n* = 2), severe oophoritis (*n* = 1), and soft tissue infection related to Fournier's gangrene (*n* = 1). The median intensive care unit (ICU) and hospital lengths of stay were 16 (IQR: 6–39) and 28.5 (IQR: 18–78) days, respectively. One patient died within 90 days (16%).

**Conclusion:**

Pneumoperitoneum with fluid collection on CT does not invariably indicate bowel perforation. In clinically stable patients with prolonged symptoms, a brief period of careful observation and reassessment may be appropriate. These findings underscore the importance of integrating radiologic features with clinical judgment to guide surgical decision-making.

## 1 Introduction

Gastrointestinal perforation (GIP) is a surgical emergency in acute care surgery (ACS) and may rapidly progress to peritoneal contamination, sepsis, and multi-organ failure (1, 2). Despite advances in antimicrobial therapy and perioperative critical care, the mortality rate is substantial, ranging from 20% to 30%, and timely operative source control remains the cornerstone of management (1).

Therefore, accurate preoperative diagnosis is essential. To guide decision-making, clinical examination, laboratory markers, and imaging findings are integrated in the emergency setting. In this regard, computed tomography (CT) has emerged as the principal diagnostic modality for suspected GIP, with high sensitivity for detecting pneumoperitoneum and intra-abdominal fluid, and for localizing the site of perforation (3, 4). Nevertheless, pneumoperitoneum identified on CT does not always indicate bowel perforation. Various conditions, including sealed microperforations, inflammatory processes, or extraabdominal sources, may also demonstrate similar radiologic findings, thus causing diagnostic uncertainty (3, 5–9).

When clinical information is limited—such as in frail patients or those with altered mental status—surgeons may adopt a lower threshold for emergency exploration to avoid missing a perforation. This approach, although cautious, may lead to nontherapeutic or negative surgical exploration. In fact, previous cohort studies have reported negative exploration rates of 3% in patients operated on for suspected GIP (5). Nonetheless, the clinical features and outcomes of patients with CT-confirmed pneumoperitoneum who ultimately have no perforation remain insufficiently characterized.

Therefore, we conducted a retrospective analysis of consecutive nontrauma patients who underwent emergency surgical exploration for suspected GIP based on CT-confirmed pneumoperitoneum and fluid collection but were found to have no perforation intraoperatively. Our aim was to characterize their clinical features and outcomes and highlight considerations relevant to surgical decision-making in this diagnostic context.

## 2 Methods

This retrospective observational review of electronic medical records was performed at Uijeongbu Eulji Medical Center (UEMC), a high-volume tertiary care hospital in South Korea. The study cohort included six consecutive patients with nontraumatic peritonitis who underwent emergency exploratory surgery between April 2021 and March 2025. The inclusion criteria were: (1) emergency surgical exploration performed for high clinical and radiologic suspicion of GIP with pneumoperitoneum and fluid collection on CT; and (2) absence of a perforative etiology identified intraoperatively. These patients were classified as cases of negative surgical exploration in suspected GIP. Patients with similar CT findings who were managed nonoperatively were excluded, as this study focused specifically on surgically explored cases. In all cases, intraoperative evaluation included examination of the entire gastrointestinal tract combined with adjunctive tests to determine occult perforation. Air-leak tests and methylene blue tests were conducted intraoperatively. Intraoperative endoscopy was not performed in any case. After surgery, operative findings and preoperative CT images were reviewed in a multidisciplinary conference involving attending acute care surgeons and radiologists. All patients underwent follow-up abdominal CT within 1 week postoperatively to confirm the absence of GIP and reevaluate the intra-abdominal condition.

Postoperative data included intensive care unit (ICU) length of stay, total hospital length of stay, and mortality within the 90-day postoperative period. All patient data were anonymized, and the study was approved by the institutional ethics committee (UEMC 2025-10-006). Emergency general surgery at UEMC is provided 24/7 by a dedicated ACS team managing a high volume of complex acute abdominal conditions.

## 3 Results

During the study period, 11 (1.1%) of 1,024 emergency surgical explorations (excluding appendectomies) were classified as negative surgical explorations. Five of these cases demonstrated fluid collection without radiologic evidence of free intraperitoneal air and were therefore excluded from the present analysis. The remaining six patients comprised the study cohort. Their median age was 67.5 years (IQR: 58–77), and three patients (50%) were men. All patients presented with abdominal pain, with a median symptom duration of 84 h (IQR: 72–148). On arrival to the emergency department, two patients had a systolic blood pressure of ≤90 mmHg, and one patient had a body temperature of >38 °C. The median white blood cell count was 13.2 × 10^9^/L (IQR: 8.7–17.7), the median neutrophil percentage was 89.8% (IQR: 87.5–92.0), and the median C-reactive protein level was 23.6 mg/dL (IQR: 19.8–29.7) (Table 1).

**Table 1:** Clinical characteristics and outcomes of patients with negative surgical exploration (*n* = 6).

Case	Symptom duration (h)	SBP ≤90 mmHg	Fever (>38 °C)	WBC (×10^9^/L)	CRP (mg/dL)	Positive culture	Preoperative diagnosis	Final diagnosis
1	72	No	Yes	8.3	33.6	Yes (blood)	GI perforation	Primary peritonitis
2	148`	No	Yes	8.7	1.1	No	Duodenal perforation	Sealed duodenal microperforation
3	72	No	No	17.7	21.3	Yes (peritoneal)	Small bowel perforation	UTI-related abscess
4	72	Yes	No	14.2	25.8	No	Small bowel perforation	Renal hematoma–related abscess
5	96	No	No	12.2	29.7	Yes (peritoneal)	Perforated appendicitis	Oophoritis
6	240	Yes	No	25.7	19.8	Yes (blood)	Duodenal perforation	Fournier's gangrene

Values are expressed as individual case data. SBP, systolic blood pressure; WBC, white blood cell count; CRP, C-reactive protein; ICU, intensive care unit.

Microbiological cultures were positive in four patients. *Prevotella buccae* was isolated from blood cultures in one patient, and *Escherichia coli* was identified in peritoneal fluid cultures in two patients; one of these cases additionally yielded *Proteus vulgaris* and *Morganella morganii*. The final postoperative diagnoses included one case of primary peritonitis, one case of sealed duodenal microperforation, two cases of peritoneal abscess secondary to urinary tract infection, one case of gynecologic source (severe oophoritis with abscess), and one case of soft tissue infection related to Fournier's gangrene.

The median ICU and hospital lengths of stay were 16 (IQR: 6–39) and 28.5 (IQR: 18–78) days, respectively. One patient required transfer to another tertiary center for abdominal wall reconstruction, and one patient died within 90 days postoperatively, corresponding to a 90-day mortality rate of 16%. Detailed CT imaging findings are summarized in Table 2.

**Table 2:** CT imaging findings.

Case	Air distribution	Retroperitoneal air	Focal bowel wall defect	Extraluminal oral contrast	Bowel wall thickening	Localized phlegmon/abscess	Fluid volume
1	Diffuse intraperitoneal	No	No	No	No	No	Large
2	Periduodenal, localized	Yes	No	No	Mild periduodenal	No	Small
3	Pelvic predominant	No	No	No	Pelvic small bowel	Yes (pelvic abscess)	Large
4	Diffuse intraperitoneal	No	No	No	No	Yes (renal hematoma–related)	Large
5	Diffuse intraperitoneal	No	No	No	Adnexal region Small bowel	Yes (pelvic abscess)	Large
6	Retroperitoneal predominant	Yes	No	No	No	Yes (soft tissue/necrotizing infection)	Small

### 3.1 Case 1. Primary peritonitis

**Figure 1:**
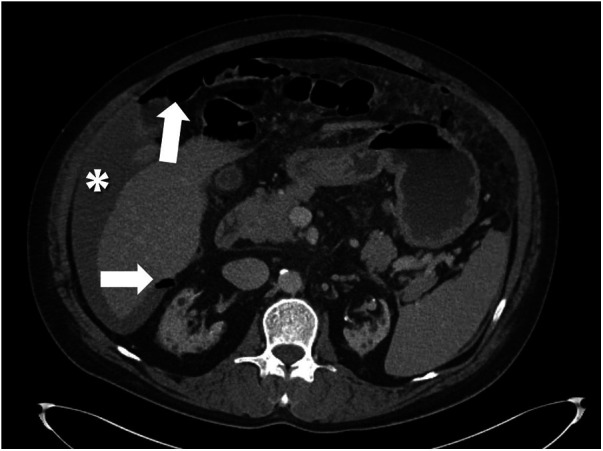
Abdominal CT findings suggestive of spontaneous bacterial peritonitis (SBP). The image reveals extensive pneumoperitoneum (arrow) and a large amount of free fluid collection (asterisk).

A patient in the late 40s with advanced chronic kidney disease receiving long-term maintenance dialysis presented with severe abdominal pain that worsened after dialysis. Contrast-enhanced CT revealed extensive pneumoperitoneum and a large volume of ascites (Figure 1), raising concern for GIP. Intraoperatively, extensive phlegmon and turbid yellow ascites were detected, but no GIP was identified. Despite irrigation and drainage, his condition rapidly progressed to sepsis and multi-organ failure; he required extracorporeal membrane oxygenation but died on postoperative day 7. Drain outputs remained persistently elevated. The probable cause of death was operative stress and severe underlying comorbidity, with long-term hemodialysis and primary peritonitis—confirmed as the possible source of the pneumoperitoneum—contributing to the fatal outcome.

### 3.2 Case 2. Sealed duodenal microperforation

**Figure 2:**
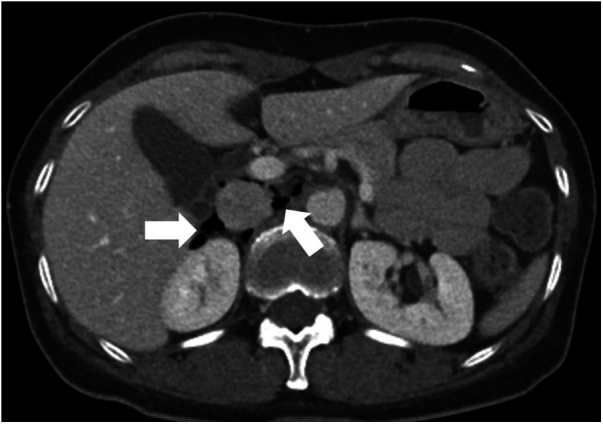
Abdominal CT findings suggestive of a spontaneously sealed duodenal microperforation. The image reveals minimal periduodenal pneumoperitoneum (asterisk).

**Figure 3:**
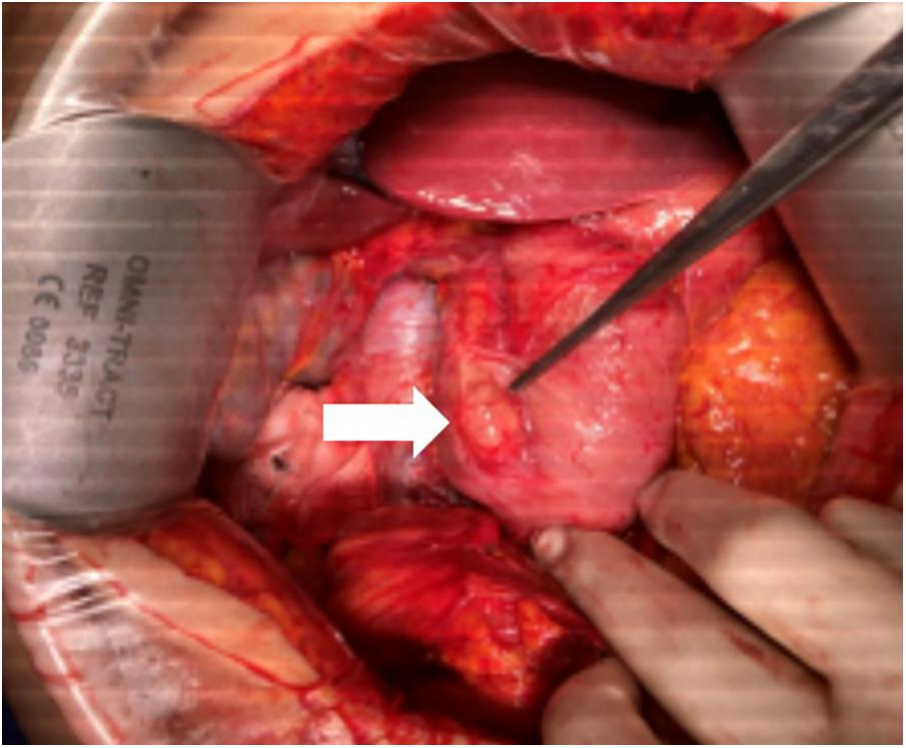
Intraoperative findings of sealed duodenal microperforation. The tip of the surgical forceps indicates the duodenal diverticulum and adjacent air bubbles (asterisk).

A patient in the 50s presented with abdominal pain for 1 week. Contrast-enhanced CT revealed retroperitoneal free air predominantly in the periduodenal region with a small adjacent fluid collection (Figure 2), highly suggestive of duodenal perforation. Emergency surgical exploration disclosed no active perforation (Figure 3), and management was limited to the placement of a surgical drain. The patient recovered uneventfully. Subsequent upper gastrointestinal (UGI) contrast study and follow-up CT scans demonstrated no enteric leakage. The final diagnosis was presumed to be a spontaneously sealed microperforation, most probably related to a preexisting lesion such as a duodenal diverticulum.

### 3.3 Case 3. UTI-related abscess

A patient in the early 70s with a history of prior urologic surgery and intermittent catheterization presented with abdominal pain for 3 days. Contrast-enhanced CT revealed pneumoperitoneum and a large volume of intraperitoneal free fluid, prompting emergency surgical exploration. Although no GIP was identified intraoperatively, extensive intra-abdominal adhesions prolonged the surgery and prompted open-abdomen management. No gastrointestinal perforation was identified intraoperatively. Dense intra-abdominal adhesions and purulent fluid were encountered. The postoperative course required additional urologic intervention and staged abdominal management. The patient survived the early postoperative period but experienced a prolonged recovery complicated by infectious events months later. The final diagnosis was secondary peritonitis associated with a genitourinary source.

### 3.4 Case 4. Renal hematoma–related abscess

**Figure 4:**
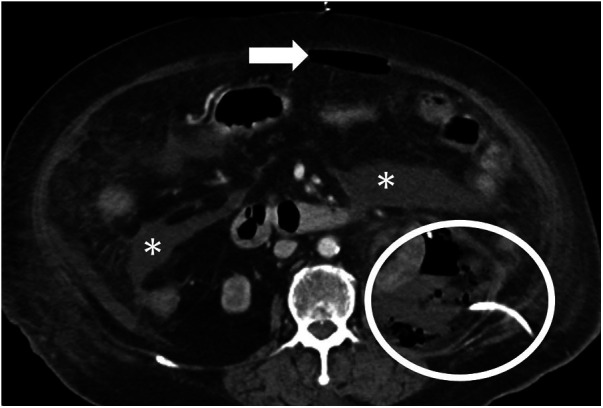
Abdominal CT findings suggestive of an intra-abdominal abscess related to an infected renal hematoma. The image reveals preexisting renal hematoma/abscess with pigtail (circle), pneumoperitoneum (arrow), and fluid collection, consistent with an abscess (asterisk).

A patient in the late 70s with multiple comorbidities presented with abdominal pain for 3 days accompanied by hypotension and altered mental status. She had recently undergone an interventional procedure for renal bleeding. Contrast-enhanced CT showed pneumoperitoneum and a large intraperitoneal fluid collection (Figure 4), prompting urgent surgical exploration. No GIP was identified; phlegmon and turbid ascites were evacuated, and multiple drains were placed. Her septic state improved rapidly after surgery. The clinical picture was believed to be most consistent with secondary peritonitis due to infection of a preexisting left renal hematoma/abscess.

### 3.5 Case 5. Oophoritis

**Figure 5:**
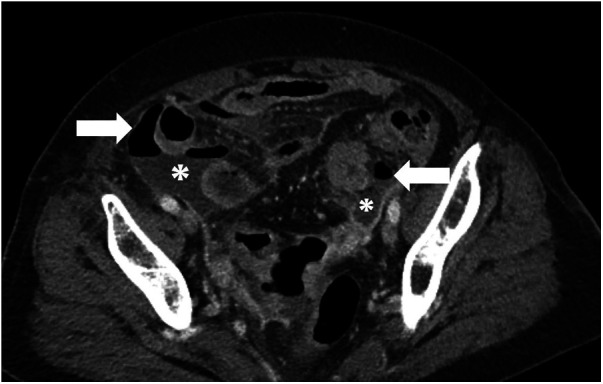
Abdominal CT findings suggestive of a pelvic abscess secondary to oophoritis. The image reveals pneumoperitoneum (arrow) and a large pelvic fluid collection, consistent with an abscess (asterisk).

**Figure 6:**
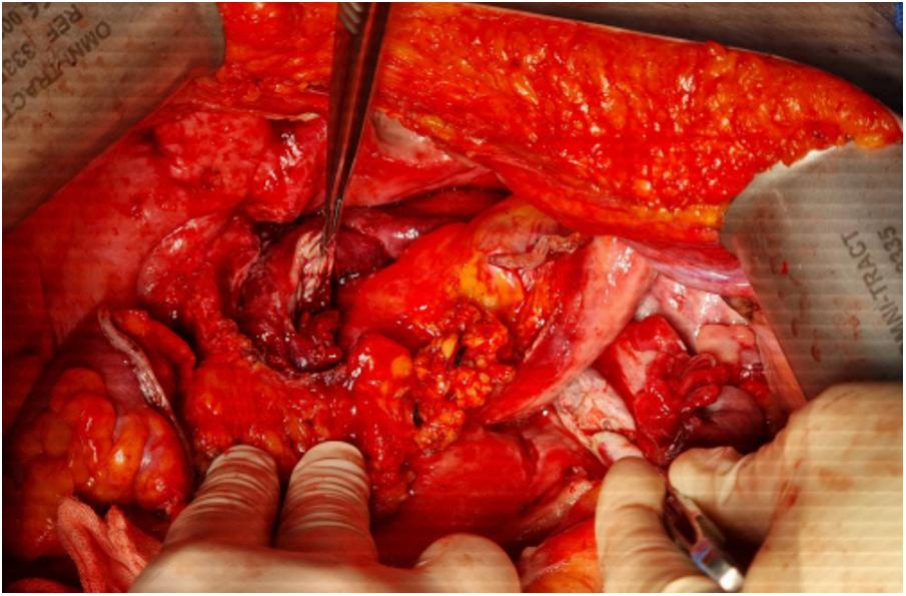
Intraoperative findings of bilateral oophoritis and resulting abscess. The tips of the surgical forceps indicate the severely inflamed and ruptured ovaries with extensive periadnexal abscess formation.

A patient in the late 70s without significant prior abdominal disease presented with several days of abdominal pain. Contrast-enhanced CT revealed marked pneumoperitoneum with a large pelvic fluid collection (Figure 5), raising a strong suspicion for colonic perforation and prompting emergency surgical exploration. Although no GIP was identified intraoperatively, severe bilateral oophoritis with extensive surrounding abscess formation was detected (Figure 6). The adjacent sigmoid colon was intact; however, the severity of local inflammation and contamination prompted a Hartmann's procedure. The postoperative diagnosis was secondary peritonitis due to severe pelvic inflammatory/infectious disease associated with oophoritis.

### 3.6 Case 6. Soft tissue infection related to Fournier's gangrene

**Figure 7:**
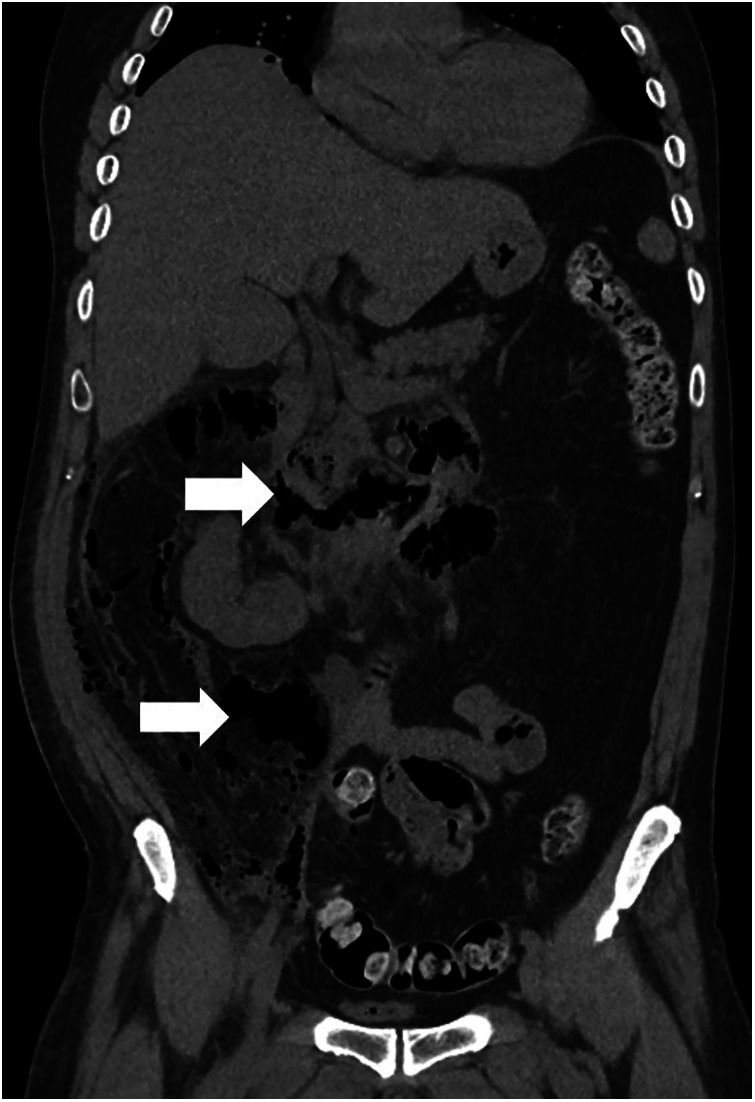
Noncontrast abdominal CT demonstrating extensive retroperitoneal free air (arrow) consistent with retroperitoneal extension of infection from necrotizing fasciitis (Fournier's gangrene).

A patient in the early 60 s presented with prolonged flank and abdominal discomfort. Noncontrast CT demonstrated a duodenal diverticulum and extensive retroperitoneal free air (Figure 7), raising high suspicion for duodenal perforation. Emergency surgical exploration using Kocher mobilization revealed no duodenal defect or enteric spillage, although foul-smelling purulent fluid was encountered. The abdomen was irrigated, and multiple drains were placed.

During the postoperative course, the patient developed necrotizing soft tissue infection requiring repeated debridement and prolonged intensive care. Secondary gastrointestinal complications occurred later in the clinical course, necessitating additional interventions. Multidisciplinary review of initial and follow-up CT imaging suggested that the initial retroperitoneal air was most consistent with the extension of necrotizing soft tissue infection rather than primary GIP. The duodenal perforation was considered a secondary event during the prolonged septic course.

## 4 Discussion

Diagnosis of acute peritonitis and the decision to proceed with emergency surgical exploration for suspected GIP remain challenging in the emergency setting. Clinical decision-making depends on the integration of symptoms, physical examination findings, and CT imaging (1). Although pneumoperitoneum accompanied by intra-abdominal fluid detected on CT is commonly considered a strong indicator of perforation (4), our findings demonstrate that such radiologic features do not invariably correspond to a perforation identified at surgery. This series emphasizes the diagnostic uncertainty that may arise when imaging findings and intraoperative assessment are discordant.

Contrast-enhanced CT is essential for evaluating acute peritonitis and provides high sensitivity for detecting free intraperitoneal air and localizing suspected perforation (3). Nevertheless, pneumoperitoneum is not synonymous with bowel perforation. Nonsurgical pneumoperitoneum accounts for an estimated 5%–15% of cases and may develop from abdominal, thoracic, gynecologic, or iatrogenic causes (3). Moreover, small or sealed perforations may present with free air without a persistent defect requiring repair. Despite advances in imaging, negative surgical exploration continues to occur, with reported rates of 3% among patients operated on for suspected perforation (5). These data suggest that CT findings, although indispensable, must be interpreted in conjunction with the overall clinical context to avoid unnecessary exploration.

In hemodynamically stable patients, careful clinical evaluation and consideration of alternative etiologies of peritonitis—such as primary peritonitis, genitourinary or gynecologic infection, or soft tissue sources—may help reduce diagnostic uncertainty. Moreover, prolonged symptom duration has been associated with negative exploration; for instance, a previous study reported that abdominal pain lasting more than 19.6 h was independently associated with this outcome (OR: 3.39, 95% CI: 1.17–9.80) (5). Therefore, in selected patients with longer symptom duration and relative physiologic stability, a brief period of close observation with serial examinations and multidisciplinary reassessment may be reasonable. When operative exploration is undertaken in equivocal cases, a minimally invasive (laparoscopic) approach may be considered.

**Figure 8:**
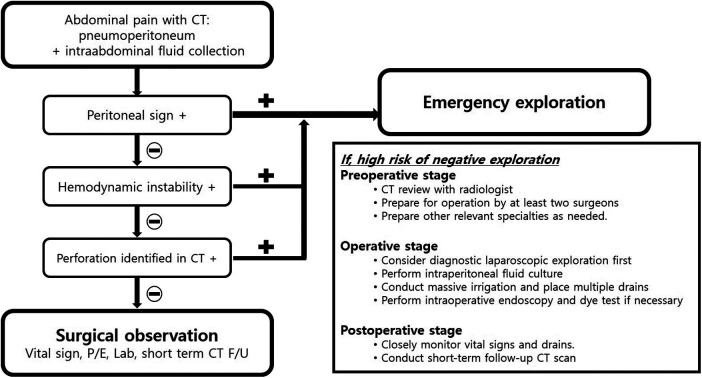
Proposed diagnostic and management pathway for suspected negative surgical exploration.

When surgical exploration fails to identify a clear perforation, adjunctive measures—such as peritoneal fluid sampling, irrigation, selective drain placement, and air-leak or dye testing—may assist in excluding occult injury. Intraoperative endoscopy may also be considered in selected cases. Postoperative clinical monitoring and follow-up imaging, once clinically stable, may further support safe management. Based on these considerations, we propose a structured decision-making algorithm for patients with ambiguous CT findings (Figure 8).

Previous studies in trauma populations have reported higher mortality rates among patients who underwent negative laparotomy than among those with confirmed injuries (10, 11). Conversely, the prognostic implications of negative surgical exploration in patients with nontraumatic acute peritonitis remain less clearly defined. The literature reports a 90-day mortality rate of approximately 6.7% among patients with confirmed GIP. In our series, one of six patients (16%) died within 90 days; nonetheless, the small sample size precludes meaningful comparison. Although surgical exploration in these cases facilitated source control and exclusion of overt perforation, the relationship between negative exploration and outcomes cannot be determined from this limited cohort. In addition, objective perioperative indices—such as serial organ failure scores and inflammatory biomarkers—were not systematically examined. Hence, these findings should be interpreted cautiously. Larger, multicenter studies with standardized outcome measures would help clarify the clinical effect of negative surgical exploration in this setting.

This study has several limitations. It was conducted at a single center and included a small number of patients, thus limiting the generalizability of the findings. Moreover, the decision to proceed with emergency surgery was often made by the attending surgeon on call, which may have introduced variability related to individual clinical judgment. Although all surgeons were experienced in acute care surgery and multidisciplinary consultation was obtained when appropriate, decision-making bias cannot be entirely excluded.

## 5 Conclusion

Pneumoperitoneum and fluid collection detected on CT does not invariably indicate bowel perforation. In clinically stable patients with prolonged symptoms, a brief period of careful observation and reassessment may be considered. This study highlights the importance of integrating radiologic findings with clinical judgment in surgical decision-making.

## Data Availability

The original contributions presented in the study are included in the article/Supplementary Material, further inquiries can be directed to the corresponding author.
